# Medical chart-reported alcohol consumption, substance use, and mental health issues in association with HIV pre-exposure prophylaxis (PrEP) nonadherence among gay, bisexual, and other men-who-have-sex-with-men

**DOI:** 10.1186/s12889-024-20934-7

**Published:** 2024-12-18

**Authors:** Paul A. Shuper, Narges Joharchi, Thepikaa Varatharajan, Isaac I. Bogoch, Mona Loutfy, Philippe El-Helou, Kevin Giolma, Kevin Woodward, Jürgen Rehm

**Affiliations:** 1 Institute for Mental Health Policy Research & Campbell Family Mental Health Research Institute, Centre for Addiction and Mental Health (CAMH), 33 Ursula Franklin St, Toronto, ON M5S 2S1 Canada; 2 Department of Psychiatry, University of Toronto, 250 College St, Toronto, ON M5T 1R8 Canada; 3 Dalla Lana School of Public Health, University of Toronto, 155 College St, Toronto, ON M5T 3M7 Canada; 4 Internal Medicine and Infectious Diseases, University Health Network, 200 Elizabeth St, Toronto, ON M5G 2C4 Canada; 5 Department of Medicine, University of Toronto, Medical Sciences Building, 1 King’s College Circle, Toronto, ON M5S 1A8 Canada; 6 Women’s College Hospital, 76 Grenville S.t, Toronto, ON M5S 1B2 Canada; 7 Maple Leaf Medical Clinic, 14 College St, Toronto, ON M5G 1K2 Canada; 8 Department of Medicine, Division of Infectious Diseases, McMaster University, 1200 Main St. W, Hamilton, ON L8N 3Z5 Canada; 9 Center for Interdisciplinary Addiction Research (ZIS), Department of Psychiatry and Psychotherapy, University Medical Center Hamburg-Eppendorf (UKE), Martinistraße 52, Hamburg, 20246 Germany; 10 Klinische Psychologie & Psychotherapie, Technische Universität Dresden’ Chemnitzer Str, 46D-01187 Dresden, Germany; 11 Department of International Health Projects, Institute for Leadership and Health Management, I.M. Sechenov First Moscow State Medical University, 8-2 Trubetskaya str, Moscow, 119991 Russian Federation; 12 Graduate Department of Community Health, Institute of Medical Science, University of Toronto, Medical Sciences Building, 1 King’s College Circle, Toronto, ON M5S 1A8 Canada

**Keywords:** HIV pre-exposure prophylaxis (PrEP), MSM, Adherence, Alcohol, Substance use, Club drugs, Mental health, Depression, Age

## Abstract

**Background:**

Although some evidence suggests that alcohol, substance use, and mental health issues diminish adherence to HIV Pre-Exposure Prophylaxis (PrEP) among gay, bisexual, and other men-who-have-sex-with-men (gbMSM), findings are somewhat inconsistent and have primarily derived from studies involving non-random samples. Medical chart extraction can provide unique insight by in part surmounting sampling-related limitations, as data for entire PrEP clinic populations can be examined. Our investigation entailed comprehensive chart extraction to assess the extent to which chart-reported alcohol, substance use, and mental health issues were associated with chart-reported PrEP nonadherence.

**Methods:**

Data from medical charts of gbMSM at two PrEP clinics in Toronto, Canada were extracted for a retrospective 12-month period (02/2018-01/2019). Charts were reviewed for all patients who were 1) ≥ 18 years old; 2) gbMSM; 3) prescribed PrEP ≥ 3 months, and 4) not in a PrEP-related drug trial. Information regarding PrEP, alcohol, substance use, mental health, and sexual behavior was extracted. PrEP adherence was classified in terms of (1) any reported nonadherence, and (2) ‘suboptimal adherence,’ reflecting nonadherence patterns indicative of insufficient pharmacological protection from HIV. Multivariate logistic regression was employed to identify factors associated with adherence outcomes.

**Results:**

Data were extracted from 4,292 clinic visits among 501 eligible patients (age: M = 39.1; duration on PrEP: M = 17.4 months; daily PrEP regimen = 93.8%). Hazardous/harmful drinking, club drug use, and mental health issues were reported among 8.8%, 22.2%, and 26.1% of patients, respectively. Any nonadherence and suboptimal adherence were reported among 37.5% and 12.4% of patients, respectively. Factors significantly associated with any nonadherence included age < 25 (AOR = 3.08, 95%CI = 1.54–6.15, *p* < .001), club drug use (AOR = 2.71, 95%CI = 1.65–4.47, *p* < .001), and condomless sex (AOR = 1.83, 95%CI = 1.19–2.83, *p* = .006). For suboptimal adherence, significant factors included age < 25 (AOR = 4.83, 95%CI = 2.28–10.22, *p* < .001), non-daily PrEP regimens (AOR = 2.94, 95%CI = 1.19–7.22, *p* = .019), missing PrEP appointments (AOR = 1.97, 95%CI = 1.09–3.55, *p* = .025), and club drug use (AOR = 1.97, 95%CI = 1.01–3.68, *p* = .033). Neither alcohol nor mental health issues were associated with nonadherence outcomes.

**Conclusions:**

Chart-indicated suboptimal adherence was present among a small subgroup of PrEP-prescribed gbMSM. Adherence-related interventions should target gbMSM who use club drugs, are younger, experience challenges attending PrEP care, and are prescribed non-daily regimens. Offering long-acting injectable PrEP when available and feasible may also improve PrEP’s HIV-preventive impact among this population.

## Introduction

Human immunodeficiency virus (HIV) remains a global health concern. In 2020, there were an estimated 37.7 million people living with HIV, including 1.5 million who acquired the virus that year alone [1]. HIV disproportionately impacts a number of key populations, including gay, bisexual, and other men who have sex with men (gbMSM), who continue to represent the highest percentage of incident HIV infections in several jurisdictions, including the United States [2], Canada [3], Australia [4], and Western Europe [5].

Among the array of strategies available to address this persistent HIV epidemic, HIV Pre-Exposure Prophylaxis (PrEP) has moved to the forefront of HIV prevention efforts that are specifically targeted toward those at risk of HIV acquisition. In most jurisdictions, PrEP typically entails taking a daily oral dose of tenofovir disoproxil fumarate/emtricitabine (TDF/FTC) or tenofovir alafenamide/emtricitabine (TAF/FTC), which substantially reduces the likelihood of contracting HIV [6–12]. Despite PrEP’s demonstrated effectiveness, regimen adherence remains crucial to the HIV-preventive success of oral-based PrEP regimens [7, 9, 10, 13–15]. Although long-acting injectable (LAI) formulations of PrEP have recently come to market [16], thereby surmounting the need for daily oral adherence, their use has yet to become universal, potentially due to barriers involving cost, availability, concerns over side effects, frequent in-person clinic visits (i.e., to receive injections), and implementation-related issues [17–19]. As a result, adhering to a daily oral PrEP regimen remains a necessity for many of those who select PrEP as an HIV-preventive strategy.

Issues involving alcohol, substance use, and mental health are common among gbMSM [20–25] and may impact one’s ability and motivation to adhere to PrEP. Although supportive evidence for associations between these issues and low levels of PrEP adherence has been yielded, findings have not been entirely consistent; thus highlighting potential nuances regarding the manner and extent to which these issues potentially hinder PrEP-taking efforts. For example, while a number of studies have shown significant links between alcohol use and PrEP nonadherence [26–29], others have demonstrated that adherence to PrEP can indeed be achieved in the context of alcohol use [30–33]. Importantly, however, these discrepant findings may derive from study-specific alcohol use categorizations that have differed in terms of consumption amounts and patterns; suggesting that significant associations between alcohol use and PrEP non-adherence may only emerge when the level of use is pronounced [29]. Associations involving the use of substances have similarly been mixed, although here too has a relatively consistent pattern emerged. Specifically, significant links with PrEP nonadherence have tended to be yielded when focusing on substances that are classified as “club drugs” (e.g., methamphetamine, ecstasy, gamma hydroxybutyrate (GHB) [31, 34, 35]) or stimulants (e.g., methamphetamine, cocaine [29, 32, 34–36]), but not when examining substances that fall outside of these categories (e.g., cannabis [29, 31, 35]). Finally, investigations examining mental health issues, which have almost exclusively focused on depression, have yielded considerably inconsistent associations. In this regard, depression has been significantly linked to both decreased [37–39] and increased [40] levels of PrEP adherence, whereas in other instances, it has been shown to be unrelated to adherence outcomes [32, 39]. Evidence suggests, however, that these disparate findings may in part be accounted for by the severity of one’s depression [41], as well as underlying sex- and gender-related dynamics [39, 40].

While these adherence-related associations involving alcohol, substance use, and mental health issues are informative for the delivery and maintenance of PrEP care, their derivation from studies primarily involving non-random samples may to some extent limit their generalizability to broader corresponding populations of PrEP-prescribed gbMSM, as participation in these initiatives would have included only those individuals who were both motivated and able to take part. Within this regard, those experiencing alcohol, substance use, and mental health issues may have been underrepresented among such convenience samples, with lower participation rates resulting from concerns over disclosing one’s issues to non-clinic personnel (e.g., external research team members), as well as having fewer opportunities of being approached for clinic-based studies given their generalized lower engagement in care [42–44].

Medical chart extraction in part surmounts these limitations, as it does not require the active participation of patients, and instead allows for the acquisition of data for *all* PrEP-prescribed gbMSM at designated PrEP-delivery sites. Although chart extraction is still subject to patient disclosure-related issues (e.g., social desirability) as well as provider querying-related limitations (e.g., having insufficient time to appropriately assess addictions- and mental health-related issues), it can serve as a direct metric of the frequency with which issues involving alcohol, substance use, mental health, and PrEP adherence are identified in a clinical setting. This latter aspect holds particular clinical relevance, as only those alcohol-, substance use-, mental health-, and adherence-related issues that are identified are likely to be addressed within their respective care-delivery site.

Recognizing these benefits, the present investigation entailed the extraction of comprehensive retrospective medical chart data for the entire population of PrEP-prescribed gbMSM at two large PrEP-delivery clinics. Using this methodology, we sought to assess the extent to which chart-indicated alcohol-, substance use-, mental health-related issues; as well as patient characteristics and PrEP-related factors, were associated with PrEP nonadherence.

## Methods

### Study population and setting

Data from medical charts of PrEP-prescribed gbMSM were extracted for a retrospective 12-month period (pre-Covid-19 pandemic; February 1st, 2018 to January 31st, 2019) at two PrEP clinics in downtown Toronto, Canada – one entailing a tertiary hospital-based outpatient clinic specializing in care for those living with or at risk for HIV, and the other a primary care community practice specializing in gbMSM- and HIV-focused treatment and prevention services. To be included in the extraction, patients had to meet the following chart-indicated criteria: 1) ≥ 18 years of age (at entry into the observation period); 2) gbMSM; 3) on PrEP for at least 3-months during the retrospective follow-up period, and 4) not involved in a PrEP-related drug trial.

### Data collection and measures

This investigation constituted a primary component of the formative research phase of an alcohol-, substance use-, and mental health-focused randomized controlled intervention trial involving PrEP-prescribed gbMSM (ClinicalTrials.gov Identifier: NCT05097430). A chart abstraction template was employed to guide and record the extraction of available demographic and clinical information from electronic and paper medical records. All procedures were approved by Research Ethics Boards at the Centre for Addiction and Mental Health (Protocol# 101–2018) and the University Health Network (Protocol# 18-5014). The following measures were included:

*Demographics.* Age (as of the start of the 12-month review period), sex/gender, and sexual behavior classification (e.g., MSM) were recorded.

*PrEP Care.* Date of initial PrEP prescription, PrEP medication type and prescribed dosing schedule (e.g., daily TDF/FTC vs. on-demand approaches), and appointment details (i.e., date and nature of clinic visit) were recorded. A missed appointment was defined as nonattendance at a scheduled PrEP-related clinic visit without making up that appointment within a subsequent 4-week period. Months of available chart data for each participant were also recorded to account for potential disparities related to the number of chart entries across participants.

*Alcohol Use.* Information regarding alcohol use, alcohol-related issues, and alcohol-related treatment or support (e.g., medication-based treatment, counselling) within the study period was extracted. Ongoing alcohol-related issues that were indicated in patients’ charts, including ones that did not specifically emerge within the 12-month period, were also categorized in terms of the patient having that issue; provided that there was no indication that the issue had been resolved. Based on these data, patients were categorized in terms of “non-,” “low-risk,” “hazardous,” or “harmful” drinking in accordance with the World Health Organization’s (WHO) guidelines [45, 46] and the Canadian Centre on Substance Use and Addiction’s (CCSA) Guidelines for Low-Risk Drinking [47]. Patients who reported not consuming any alcohol were included in the non-drinking category. Low-risk drinking was defined as fulfilling one of the following drinking patterns: no more than 15 drinks a week, with no more than 3 drinks a day on most days or up to 4 drinks on a single occasion. Hazardous drinking reflected patterns of consumption that included 15 to 28 drinks per week, 5–7 drinks on a single occasion, one drink while driving or operating machinery, and/or drinking alcohol while using substances. Harmful drinking was defined as engaging in one or more of the following patterns: >28 drinks per week, > 7 drinks on a single occasion, driving or operating machinery while having at least two drinks, and/or consuming more than four drinks while using a substance. Indications of alcohol use disorder relapse, addiction, and heavy drinking in the charts were also classified as harmful drinking. As only a limited number of patients were classified within each risk group, the sample was aggregated into non-hazardous (i.e., non-drinking and low-risk drinking) and hazardous drinking (i.e., hazardous and harmful drinking) categories.

*Substance Use.* Indications of substance use, substance use-related issues, and/or substance use treatment (e.g., medication-based treatment, counselling) during the past 12 months were recorded. Similar to alcohol consumption, ongoing substance use issues that were indicated in patients’ charts, including those that did not specifically emerge during the 12-month period, were categorized in terms of the patient having that issue; provided that there was no indication that the issue had been resolved. Based on these data, “any substance use” was defined as the recorded use of one or more of the following drugs: cannabis, cocaine, poppers, methamphetamine, 3,4-methyl​enedioxy​methamphetamine (MDMA)/ecstasy, gamma hydroxybutyrate (GHB), or hallucinogens. Furthermore, given the category-specific substance use patterns demonstrated in past research, the use of “club drugs” was also examined, which was defined in accordance with the National Institute on Drug Abuse’s classification and included any recorded use of methamphetamines, ecstasy/MDMA, GHB, or hallucinogens [48].

*Mental Health.* An individual was considered to be experiencing a mental health issue within the study period if there were corresponding chart indications pertaining to a mental illness diagnosis (e.g., depression, anxiety, bipolar disorder), a prescription for a medication used to treat symptoms of mental illness (e.g., bupropion, lorazepam, risperidone), or a referral for treatment of a mental illness (e.g., psychiatrist, counsellor, social worker).

*Sexual Behaviors and Sexually Transmitted Infections.* Information pertaining to the engagement in condomless sex was extracted from clinic notes for the 12-month retrospective period. Individuals were classified as those whose charts indicated the engagement in any condomless anal sex (either insertive or receptive) versus those without such indication. Individuals were also classified based on the presence or absence of any chart-reported sexually transmitted infections (STI) (i.e., chlamydia, gonorrhea, syphilis).

*Study Outcomes: PrEP Nonadherence.* Two different outcomes related to PrEP nonadherence were generated based on available chart data, and were operationalized in terms of (1) any nonadherence, and (2) suboptimal adherence. Any nonadherence was based on a very broad definition and identified patients who had chart-based indications of missing *any* of their PrEP doses within the study period. Experiencing any nonadherence included chart indications that ranged from missing only a minimal number of PrEP doses (e.g., “missed 1 dose last month,” “misses 1 dose per week,” “missed a tablet from time to time”) to more pronounced levels of nonadherence (e.g., “missed 3 in a row,” “not optimal,” “nonadherent,” “being on and off PrEP,” or “infrequent use of PrEP”). In contrast, suboptimal adherence, a more stringent measure, identified individuals who had a nonadherence pattern indicative of being insufficiently pharmacologically protected from HIV. For example, individuals whose chart entries stated “missed 3 in a row,” “not optimal,” “nonadherent,” “being on and off PrEP,” or “infrequent use of PrEP” were categorized as being suboptimally adherent, whereas individuals who “missed 1 dose last month” were not. Based on the operationalization of our outcomes, all patients who were classified as suboptimally adherent were also classified as experiencing any nonadherence, but not vice versa. As detailed in the next section, the two outcomes were evaluated separately in our analyses.

### Statistical analyses

Descriptive statistics were used to summarize patient characteristics and PrEP-related factors. Study variables were dichotomized for inferential analyses, where scores of “0” and “1” reflected the absence or presence of an indicator/condition at any point during the observation period respectively (e.g., no club drug use = 0, club drug use = 1). When no information regarding a specific indicator/condition was present in a patient’s chart, a score of “0” was given. For example, if a patient’s chart contained no reference to either any mental health issue, medication for a mental health issue, or referral for mental health treatment, the individual received a score of “0” for the mental health factor.

The primary outcomes - any nonadherence and suboptimal adherence - were coded as “0 = adherent” and “1 = nonadherent”/“suboptimally adherent.” For charts that did not contain information pertaining to adherence, a score of “0” was assigned to reflect that no adherence-related issues were present.

Statistical analyses were cross-sectional, wherein a chart-based report regarding a specific factor at *any* point during the 12-month observation period (e.g., club drug use indicated at any visit during the period) was evaluated for its association with a study outcome (e.g., any nonadherence) that had also been reported at *any* point during the observation period. Univariate logistic regression was employed to identify associations between chart-indicated factors and each adherence outcome. Factors that demonstrated an association in the univariate analyses at the *p* < .10 significance level were included in corresponding, outcome-specific multivariate logistic regression models. The exception to this inclusion rule pertained to factors exhibiting small cell sizes and/or collinearity, which included, for example, some specific substances (e.g., small n for GHB) and mental health conditions (e.g., small n for borderline personality disorder; significant correlation between depression and anxiety); leading to the inclusion of only the more encompassing factor (i.e., club drug use and the experience of any mental health issue, respectively). Based on these criteria, factors included in the multivariate logistic regression model for any nonadherence entailed age <25, being on PrEP <12 months, months of available chart data, cocaine use, club drug use, experiencing any mental health issue, and engaging in condomless anal sex. For suboptimal adherence, factors in the model included age <25, taking a non-daily PrEP regimen, missing any PrEP appointments, hazardous alcohol use, popper use, club drug use, and experiencing any mental health issue. All analyses were conducted using SPSS Version 26.0 [49].

## Results

Across the two clinics, over 700 patient charts from 27 PrEP clinicians were reviewed for eligibility, of which 501 met eligibility criteria. Data were extracted from 4,292 clinic visits, comprised of 2,436 (56.8%) PrEP-related visits (e.g., PrEP consultation, PrEP follow-up, STIs), 714 (16.6%) laboratory testing visits, 94 (2.2%) visits that focused on mental health and substance use issues, and 1,048 (24.4%) visits related to other medical concerns (e.g., influenza vaccination, bodily pain). As shown in Table 1, the mean age of patients was 39.1 (SD = 10.8, range = 18–76), and virtually all had their sex/gender recorded as “male” (*n* = 499, 99.6%), with “transman” indicated for two patients (0.4%). The majority of patients (*n* = 473, 94.4%) were indicated as MSM or as having sex with men only, while a smaller number (*n* = 28, 5.6%) were indicated as having sex with both men and women. Race/ethnicity was not indicated for 91% of patients and is therefore not reported.

**Table 1:** Chart-reported characteristics of gay, bisexual, and other men-who-have-sex-with-men who were receiving PrEP (*N* = 501)

Characteristic	*n* (%)
**Age**	
Mean (SD, Range)	39.10 (10.74, 18–76)
< 25 years old	41 (8.2)
**Sex/Gender**	
Male	499 (99.6)
“Transman”	2 (0.4)
**Sexual behavior classification**	
MSM/sex with men only	473 (94.4)
Sex with men and women	28 (5.6)
**Duration on PrEP**	
Mean (SD, Range)	17.4 (14.5, 3–88)
< 12 months	253 (50.5)
**PrEP regimen**	
Daily PrEP only	470 (93.8)
Non-daily PrEP regimen	31 (6.2)
Type of non-daily PrEP regimen:	
On demand PrEP	2 (0.4)
Intermittent PrEP	6 (1.2)
Transitioned between regimens (e.g., daily/on-demand/intermittent)	23 (4.6)
Hazardous/harmful alcohol consumption	44 (8.8)
Substance use (any)	165 (32.9)
Use of specific substances:	
Cannabis	55 (11.0)
Cocaine	35 (7.0)
Poppers	30 (6.0)
Club Drugs	111 (22.2)
Use of specific club drugs:	
Methamphetamine	89 (17.8)
MDMA/Ecstasy	25 (5.0)
GHB	11 (2.2)
Hallucinogens	2 (0.4)
Mental health issue (any)	131 (26.1)
Specific mental health issues:	
Depression	96 (19.2)
Anxiety disorder	91 (18.2)
Bipolar disorder	4 (0.8)
Borderline personality disorder (BPD)	1 (0.2)
Obsessive compulsive disorder (OCD)	4 (0.8)
Post-traumatic stress disorder (PTSD)	7 (1.4)
**Sexual behavior indicators**	
Condomless anal sex	339 (67.7)
Sexually transmitted infection (STI)	193 (38.5)
**PrEP adherence**	
Any nonadherence	188 (37.5)
Suboptimal adherence	62 (12.4)

PrEP: Pre-Exposure Prophylaxis; MSM: Men who have sex with men; MDMA: 3,4-methyl​enedioxy-​methamphetamine; GHB: gamma hydroxybutyrate

Note The denominator for all calculated percentages is 501

### PrEP history, regimen, and Follow-Up care

The average duration on PrEP was 17.4 months (SD = 14.5, range = 3–88). All patients had been prescribed oral emtricitabine/tenofovir disoproxil fumarate, and the vast majority were on a daily regimen (470, 93.8%), with smaller proportions taking PrEP on-demand (0.4%), intermittently (1.2%), or transitioning between daily, on-demand, and/or intermittent regimens (4.6%). A quarter of the sample (*n* = 125, 25.0%) missed one or more of their PrEP appointments, and 25 patients (5.0%) terminated PrEP care during the follow-up period.

### Alcohol, substance use, and mental health issues

An indication of past year hazardous/harmful drinking was found among 8.8% of the sample. One-third of the sample (32.9%) had an indication of any past year substance use, and more than one-fifth (22.2%) had reports of using club drugs in the past year. More than one quarter of the study population (26.1%) was identified as experiencing a mental health issue within the study period, with depression (19.2%) and anxiety (18.2%) being the most frequently reported issues.

### Any nonadherence: prevalence and correlates

More than a third of the sample (*n* = 188/501, 37.5%) had chart-based indications of any nonadherence. Results from univariate and multivariate logistic regression analyses assessing factors associated with any nonadherence can be found in Table 2. As shown in the Table, multivariate analyses demonstrated that age < 25 (AOR = 3.08, 95%CI = 1.54–6.15, *p* < .001), club drug use (AOR = 2.71, 95%CI = 1.65–4.47, *p* < .001), and the engagement in condomless sex (AOR = 1.83, 95%CI = 1.19–2.83, *p* = .006) were significantly associated with any nonadherence.

**Table 2:** Correlates of any nonadherence: Univariate and multivariate logistic regression

Factor	Adherent (*n* = 313) *n* (%)	Nonadherent (*n* = 188) *n* (%)	OR (95% CI)	*p*	AOR (95% CI)	*p*
Age: < 25 years*	15 (4.8)	26 (13.8)	3.19 (1.64–6.19)	< 0.001	3.08 (1.54–6.15)	< 0.001
On PrEP < 12 months*	143 (45.7)	110 (58.5)	1.68 (1.16–2.42)	0.006	1.42 (0.70–2.90)	0.331
Months of available chart data [Mean(SD)]*	9.22 (3.42)	8.31 (3.44)	0.93 (0.88–0.98)	0.004	1.05 (0.94–1.17)	0.401
PrEP regimen type = non-daily	19 (6.1)	12 (6.4)	1.06 (0.50–2.23)	0.888		
Missed any PrEP appointments	84 (26.8)	41 (21.8)	0.76 (0.50–1.17)	0.209		
Hazardous alcohol use	24 (7.7)	20 (10.6)	1.43 (0.77–2.67)	0.257		
Substance use (any)	82 (26.2)	83 (44.1)	2.23 (1.52–3.27)	< 0.001		
Use of specific substances:						
Cannabis	31 (9.9)	24 (12.8)	1.33 (0.76–2.35)	0.322		
Cocaine*	17 (5.4)	18 (9.6)	1.84 (0.93–3.67)	0.082	1.24 (0.58–2.64)	0.577
Poppers	17 (5.4)	13 (6.9)	1.29 (0.61–2.73)	0.499		
Club Drugs*	47 (15.0)	64 (34.0)	2.92 (1.90–4.50)	< 0.001	2.71 (1.65–4.47)	< 0.001
Use of specific club drugs:						
Methamphetamine	39 (12.5)	50 (26.6)	2.55 (1.60–4.06)	< 0.001		
MDMA/Ecstasy	12 (3.8)	13 (6.9)	1.86 (0.83–4.17)	0.130		
GHB	3 (1.0)	8 (4.3)	4.59 (1.20-17.53)	0.026		
Hallucinogens	1 (0.3)	1 (0.5)	1.67 (0.10-26.83)	0.718		
Mental health issue (any)*	91 (29.1)	40 (21.3)	0.66 (0.43–1.01)	0.055	0.68 (0.43–1.07)	0.096
Specific mental health issues:						
Depression	66 (21.0)	30 (16.0)	0.71 (0.44–1.14)	0.159		
Anxiety disorder	63 (20.1)	28 (14.9)	0.69 (0.43–1.13)	0.143		
Bipolar disorder	2 (0.6)	2 (1.1)	2.52 (0.42–15.23)	0.313		
Borderline personality disorder (BPD)	1 (0.3)	0 (0.0)	1.00 (0.00–0.00)	1.000		
Obsessive compulsive disorder (OCD)	3 (1.0)	1 (0.5)	0.55 (0.06–5.35)	0.609		
Post-traumatic stress disorder (PTSD)	6 (1.9)	1 (0.2)	0.27 (0.33–2.29)	0.232		
Sexual behavior indicators						
Condomless anal sex*	193 (61.7)	146 (77.7)	2.16 (1.43–3.26)	< 0.001	1.83 (1.19–2.83)	0.006
Sexually transmitted infection (STI)	117 (37.4)	76 (40.4)	1.14 (0.79–1.65)	0.498		

*Included in the multivariate model. OR: Odds Ratio; AOR: Adjusted Odds Ratio; PrEP: Pre-Exposure Prophylaxis; MSM: Men who have sex with men; MDMA: 3,4-methyl​enedioxymethamphetamine; GHB: gamma hydroxybutyrate

### Suboptimal adherence: prevalence and correlates

Suboptimal adherence was identified among 12.4% of the sample (*n* = 62/501). Results from univariate and multivariate logistic regression analyses assessing factors associated with suboptimal adherence can be found in Table 3. As shown in the Table, multivariate analyses demonstrated that age < 25 (AOR = 4.83, 95%CI = 2.28–10.22, *p* < .001), taking a non-daily PrEP regimen (AOR = 2.94, 95%CI = 1.19–7.22, *p* = .019), missing any PrEP appointments (AOR = 1.97, 95%CI = 1.09–3.55, *p* = .025), and club drug use (AOR = 1.97, 95%CI = 1.01–3.68, *p* = .033) were significantly associated with suboptimal adherence.

**Table 3:** Correlates of suboptimal adherence: Univariate and multivariate logistic regression

Factor	Adherent (*n* = 439) *n* (%)	Suboptimally adherent (*n* = 62) *n* (%)	OR (95% CI)	*p*	AOR (95% CI)	*p*
Age: < 25 years*	27 (6.2)	23 (22.6)	4.45 (2.19–9.07)	< 0.001	4.83 (2.28–10.22)	< 0.001
On PrEP < 12 months	220 (50.1)	33 (53.2)	1.13 (0.67–1.93)	0.647		
Months of available chart data [M(SD)]	8.88 (3.47)	8.83 (3.35)	1.00 (0.92–1.08)	0.901		
PrEP regimen type = non-daily*	23 (5.2)	8 (12.9)	2.68 (1.14–6.29)	0.024	2.94 (1.19–7.22)	0.019
Missed any PrEP appointments*	102 (23.2)	23 (37.1)	1.95 (1.11–3.41)	0.020	1.97 (1.09–3.55)	0.025
Hazardous alcohol use*	35 (8.0)	9 (14.5)	1.96 (0.89–4.30)	0.094	1.71 (0.72–4.02)	0.223
Substance use (any)	136 (31.0)	29 (46.8)	1.96 (1.14–3.35)	0.014		
Use of specific substances:						
Cannabis	46 (10.5)	9 (14.5)	1.45 (0.67–3.13)	0.343		
Cocaine	29 (6.6)	6 (9.7)	1.52 (0.60–3.81)	0.378		
Poppers*	21 (4.8)	9 (14.5)	3.38 (1.47–7.76)	0.004	2.41 (0.97–5.98)	0.058
Club Drugs*	89 (20.3)	22 (35.5)	2.16 (1.22–3.82)	0.008	1.97 (1.01–3.68)	0.033
Use of specific club drugs:						
Methamphetamine	78 (16.6)	11 (25.8)	1.74 (0.94–3.25)	0.080		
MDMA/Ecstasy	19 (4.3)	6 (9.7)	2.37 (0.91–6.18)	0.078		
GHB	6 (1.4)	5 (8.1)	6.33 (1.87–21.41)	0.003		
Hallucinogens	1 (0.2)	1 (1.6)	7.18 (0.44-116.28)	0.165		
Mental health issue (any)*	109 (24.8)	22 (35.5)	1.67 (0.95–2.93)	0.076	1.62 (0.88–2.97)	0.119
Specific mental health issues:						
Depression	58 (13.2)	12 (19.4)	1.72 (0.94–3.17)	0.080		
Anxiety disorder	53 (12.1)	12 (19.4)	1.69 (0.91–3.14)	0.098		
Bipolar disorder	3 (0.7)	2 (3.2)	4.84 (0.79–29.59)	0.087		
Borderline personality disorder (BPD)	1 (0.2)	0 (0.0)	--	--		
Obsessive compulsive disorder (OCD)	3 (0.7)	1 (1.6)	2.38 (0.24–23.27)	0.455		
Post-traumatic stress disorder (PTSD)	7 (1.6)	0 (0.0)	--	--		
Sexual behavior indicators						
Condomless anal sex	294 (67.0)	45 (72.6)	1.31 (0.72–2.36)	0.378		
Sexually transmitted infection (STI)	170 (38.7)	23 (37.1)	0.93 (0.54–1.62)	0.805		

*Included in the multivariate model. OR: Odds Ratio; AOR: Adjusted Odds Ratio; PrEP: Pre-Exposure Prophylaxis; MSM: Men who have sex with men; MDMA: 3,4-methyl​enedioxymethamphetamine; GHB: gamma hydroxybutyrate; “--”: could not be calculated due to “0” cell

## Discussion

Extraction of medical chart data from over 4,000 clinic visits was conducted for a sample comprised of all gbMSM PrEP patients at two large PrEP-delivery sites. While a considerable number of these patients (i.e., 37.5%) were identified as not being completely adherent to their PrEP regimen, a relatively smaller subgroup (i.e., 12.4%) had indications of PrEP adherence levels that put them at risk for acquiring HIV.

Consistent with previous survey-based investigations [31, 34], patients for whom club drug use was chart-reported were significantly more likely than those without such a report to have indications of both any nonadherence and suboptimal adherence. Proximally, being under the influence of a club drug such as methamphetamine can hinder one’s ability to remember to take one’s medication at a coinciding dosing time [50]. Distally, the use of such substances may impair neurocognitive functioning [51], which can detract from the planning and execution of the continuum of behaviors necessary for successful adherence (e.g., attending PrEP appointments, obtaining prescription refills, devising and maintaining a dosing schedule). Club drug use may also be indicative of disrupted lifestyles that in-and-of-themselves complicate ongoing medication-taking efforts [52]. Based on the consistency in associations between club drug use and nonadherence found in the present as well as past research [31, 34], tailored, behaviorally-focused adherence support interventions should be offered to PrEP-prescribed gbMSM who seek to maximize the HIV-preventive benefits of oral-based PrEP regimens within the context of their club drug use. Offering club drug-focused harm-reduction programs to those who seek to reduce their substance use may also prove to be effective for enhancing PrEP adherence (see [53] for a related example). Alternatively, when available, feasible, and acceptable, LAI forms of PrEP, which are not reliant on daily regimen adherence, should be strongly considered for this subpopulation [31, 35].

Unlike club drug use, alcohol was not significantly associated with adherence outcomes. Although this finding adds to the somewhat inconsistent alcohol/PrEP adherence patterns demonstrated in the extant literature [26–33], it should be noted that in the present investigation, (1) only minimal information pertaining to alcohol consumption was available in the charts, and (2) hazardous and harmful alcohol use were indicated very infrequently. Of further importance is that a confidential, self-report survey conducted with a subsample of the present chart extracted population yielded a markedly higher prevalence of hazardous/harmful alcohol use (i.e., 31.9%) [29] vs. what was identified through extraction (i.e., 8.8%). Based on this discrepancy, the present chart data suggest that alcohol consumption was potentially (1) under-disclosed by patients to their providers; (2) queried, identified, or recorded to a limited extent by providers; or (3) some combination of both. This, in turn, limits the ability to generate definitive conclusions regarding the non-significant, chart-derived alcohol/PrEP adherence association that emerged.

Similar to the alcohol findings, mental health issues were not significantly associated with adherence outcomes. Tests of similar associations in past research, primarily involving depression, have shown a considerable degree of inconsistency [32, 37–40]; suggesting that the association between such factors and PrEP adherence may be non-linear and even bidirectional in nature [40, 43]. Despite the lack of a demonstrated association in the current initiative, the high prevalence of chart-indicated mental health issues highlights the imperative of making mental health services and treatment readily available to this population.

Compared to their older counterparts, gbMSM under the age of 25 had three times greater odds of having a chart-based indication of any nonadherence, and almost five times greater odds of being identified as suboptimally adherent. These marked associations accord with findings from previous studies [38, 54–60]; and may be reflective of the supposition that younger gbMSM are comparatively less adherent to their PrEP regimens as a result of relatively less well-developed cognitive planning and organizational capabilities; increased vulnerability to peer influence; and a lower perceived risk of HIV acquisition [61]. Accordingly, concerted efforts involving enhanced treatment support for younger PrEP-prescribed gbMSM, such as increasing the frequency of PrEP clinic appointments [61], and offering mobile/electronic interventions [62], are clearly warranted to ensure adequate protection from HIV among this group.

PrEP-related factors, involving missing one’s PrEP appointments and the prescription of a non-daily PrEP regimen, were also significantly associated with suboptimal adherence. Missing appointments may be reflective of underlying organizational challenges (e.g., the ability to plan and maintain a schedule of events) that similarly affect one’s ability to adhere to PrEP. Additionally, in some circumstances, PrEP appointment attendance may be a requisite for obtaining a prescription refill, and failing to attend would therefore result in the absence of pills to take. Regarding regimen type, increased non-adherence to non-daily vs. daily PrEP regimens may derive from the added complexities associated with remembering to take one’s medication at irregular times, including prior to sexual activity which may have been unplanned [63]. It may alternatively be the case that some patients who experience challenges adhering to daily regimens are also the ones who transition to intermittent or on-demand regimens to help better match their life circumstances or sexual behavior patterns. The extent of chart data available for the present investigation, however, precludes us from determining the reason behind regimen choice, as well as whether regimen decisions derived from the provider, the patient, or both parties. Further research is needed to better understand these non-daily PrEP users and to identify the potentially unique adherence-related challenges they experience.

Of particular relevance to PrEP-focused HIV risk-reduction efforts is that nonadherence was significantly associated with the engagement in condomless sex. “Risk compensation,” entailing a general increase in condomless sex following PrEP uptake, has been shown to occur [64–66], but its impact on increasing the likelihood of HIV seroconversion is only realized when combined with poor PrEP adherence [67]. This confluence, as exhibited in the current study, can result in situations in which PrEP-prescribed individuals are neither pharmacologically nor physically protected from HIV. While this notable HIV-risk-relevant association accords with a similar pattern reported by Newcomb et al. [64], who found that the highest rates of condomless receptive anal sex were among those on PrEP but who were nonadherent, it contrasts with the opposite pattern yielded by Pasipanodya et al. [68], who in turn suggested that PrEP-prescribed individuals may consciously align their condom use decisions with their perceived degree of PrEP-based HIV protection; which derives from their level of adherence. Accordingly, to account for and better understand these disparate findings, the concept of risk compensation, particularly in the context of PrEP adherence, very likely needs to be viewed as a dynamic rather than a static process [68]; not only across PrEP-prescribed populations, but also within PrEP-prescribed individuals themselves. In this regard, future tests of associations between PrEP adherence and the engagement in condomless sex would need to address the nature of the sexual acts engaged in (i.e., receptive vs. insertive positioning), as well as the characteristics of the partners involved, including their HIV serostatus, HIV viral load, PrEP status, and relationship type (e.g., casual vs. steady) [64, 69].

Study findings should be viewed in terms of limitations. First, factors (e.g., club drug use) and outcomes (e.g., suboptimal adherence) were classified based on their indicated *presence* in a patient’s chart. When such information was absent (e.g., no statements made about club drugs), the patient was classified as not experiencing the issue. This categorization method was therefore subject to the limitations associated with patient disclosure, including social desirability bias; provider querying; and provider documentation of these issues. As such, the values reported in the present investigation, particularly those involving potentially sensitive topics such as alcohol consumption, substance use, mental health, and missed PrEP doses, may be underestimates of the actual rates. Second, although data were extracted for up to a 12-month period for each participant, longitudinal analyses that evaluated associations between factors and outcomes were not possible, as it was uncommon for factors and outcomes to be reported at multiple time points within one’s 12-month chart history. Analyses were therefore cross-sectional in nature, wherein a report of an issue at *any* point during the 12-month period was evaluated for its association with each study outcome (i.e., any nonadherence; suboptimal adherence) that had also been reported at *any* point during the same period. As a result, definitive conclusions regarding the temporal and causal nature of the demonstrated associations cannot be made. Third, the nature and extent of chart documentation differed substantially across the 27 providers who served PrEP patients. For example, in some instances, adherence was recorded in accordance with specific questions (e.g., number of missed doses during the past week, past month), whereas in other instances, information pertaining to adherence was written in a more open-ended manner. Because patients were not randomly assigned to providers, the possibility of systematic differences in the querying and/or documentation of issues, as well as the type of patients served by each provider, may have influenced the resultant findings. Fourth, as indicated above, charts were almost entirely devoid of information pertaining to some factors (e.g., race/ethnicity), and were very limited for other key factors of interest (e.g., alcohol, specific substances). This prevented the inclusion of the former factors in the analyses, and it potentially impacted the associations involving the latter. Fifth, although the sample was large and comprehensive, chart extraction was conducted at two PrEP-delivery clinics in downtown Toronto, and the patient populations served at these two sites may not be representative of the broader population of PrEP-prescribed gbMSM. Lastly, as the sample was comprised entirely of men, results may not generalize to samples of women who have been prescribed PrEP.

## Conclusions

Although suboptimal adherence to PrEP was indicated in the medical charts of a relatively small subgroup of gbMSM, a set of clinically-relevant, readily identifiable, and predominantly modifiable factors was found to be significantly associated with adherence levels that provided insufficient pharmacological protection from HIV. Targeting adherence support efforts to PrEP-prescribed gbMSM who use club drugs, who experience challenges attending PrEP appointments, who are prescribed non-daily PrEP regimens, and who are younger, as well as offering LAI PrEP as an alternative to oral-based regimens when possible, may help improve PrEP’s HIV-preventive impact among this population.

## Data Availability

The datasets generated during and/or analyzed during the current study are not publicly available due to research ethics-related requirements but may be available from the corresponding author on reasonable request.

## Abbreviations

AOR: Adjusted odds ratio
CCSA: Canadian Centre on Substance Use and Addiction
gbMSM: Gay, bisexual, and other men-who-have-sex-with-men
GHB: Gamma hydroxybutyrate
HIV: Human Immunodeficiency Virus
LAI: Long-acting injectable
MDMA: 3,4-Methyl enedioxy methamphetamine
OR: Odds ratio
PrEP: Pre-Exposure Prophylaxis
STI: Sexually transmitted infection
TAF/FTC: Tenofovir alafenamide/emtricitabine
TDF/FTC: Tenofovir disoproxil fumarate/emtricitabine
WHO: World Health Organization
